# Thioredoxin prevents thioacetamide-induced acute hepatitis

**DOI:** 10.1186/1476-5926-2-S1-S6

**Published:** 2004-01-14

**Authors:** Hiroaki Okuyama, Yasuyuki Shimahara, Hajime Nakamura, Shinichi Araya, Norifumi Kawada, Yoshio Yamaoka, Junji Yodoi

**Affiliations:** 1 Department of Gastroenterological Surgery, Graduate School of Medicine, Kyoto University, Japan; 2 Department of Biological Responses, Laboratory of Infection and Prevention, Institute for Virus Research, Kyoto University; Shogoin, Kawahara-cho, Sakyo-ku, Kyoto, Japan 606-8397; 3 Department of Hepatology, Graduate School of Medicine, Osaka City University, Japan

## Introduction

Thioredoxin (Trx) is an endogenous multifunctional protein with a redox-active disulfide/dithiol within the conserved active site sequence: -Cys-Gly-Pro-Cys- [1]. Trx is also a stress-inducible protein whose expression is enhanced by various types of stresses, e.g., viral infection, exposure to UV light, x-ray irradiation, and hydrogen peroxide (H_2_O_2_) [2]. Furthermore, Trx is a scavenger of reactive oxygen species (ROS), and recombinant Trx has protective activity against ROS-mediated cytotoxicity [3]. We have previously reported that Trx attenuates an ischemic brain damage by scavenging radicals [4].

Based on these considerations, we hypothesized that Trx could attenuate oxidants-mediated acute hepatitis. TAA is known to be a hepatotoxin via generation of free radicals, resulting in ROS-mediated acute hepatitis. In the present study, we subjected wild type (Wt) mice and Trx transgenic (Tg) mice to TAA-induced acute lethal hepatitis. Our findings shed light on the protective role of Trx for acute liver injury.

## Methods

### TAA-induced Acute Lethal Hepatitis

Male mice weighing 25–30 g were used for *in vivo *liver injury models. We injected TAA (100 –g/g) into Trx Tg mice (n = 16) and Wt mice (n = 16). We observed the survival rate of TAA-treated mice until 7 days. Moreover, to estimate the pathophysiological values of livers from Wt (n = 9) and Tg mice (n = 9), twenty-four hours after TAA administration, mice were anesthetized by diethylether, and the livers were removed.

## Results

### Prevention of acute lethal hepatitis in Tg mice

We used Tg mice to check the protective role of Trx for acute hepatitis. We subjected both Wt and Tg mice to TAA-induced acute lethal hepatitis. Survival rate after TAA administration was significantly higher in Tg mice (n = 16) than in Wt mice (n = 16) (P &lt; 0.01). Twenty four hours after TAA administration, the AST and ALT levels were significantly lower in Tg mice than in Wt mice (AST; 7,930 U/ml in Wt mice vs 1,417 U/ml in Tg mice, P &lt; 0.01, ALT; 10,933 U/ml in Wt mice vs 1,885 U/ml in Tg mice, P &lt; 0.01). Histological analysis by Hematoxyline & Eosin staining showed that the destruction of hepatic sinusoid with massive thrombosis was prominent in wt mice, whereas it was observed slightly in Tg mice.

### Prevention of apoptosis in Tg mice

We found that TAA (100 –g/g) induces apoptosis in the liver of Wt mice. To determine whether Trx inhibits TAA-induced apoptosis in the liver, we checked the extent of apoptotic cells by TUNEL staining and DNA laddering assay. TUNEL-positive cells around the hepatic central vein of the livers were smaller in number in Tg mice than in Wt mice. DNA laddering was striking in TAA-treated livers of Wt mice, whereas it was hardly detected in TAA-treated livers of Tg mice.

## Discussion

Trx has not only anti-oxidant effect but also anti-apoptotic effect. We have previously reported that Trx inhibits brain ischemic injury via anti-oxidative effect [4]. We have also reported that Trx inhibited alcohol-induced hepatocyte cell death [5]. The present study showed that Trx inhibited TAA-induced apoptosis of hepatocytes via the inhibition of cytochrome c release from mitochondria. We have previously reported that, in Jurkat T cells, a thioloxidant, diamide, induces cytochrome c release, resulting in caspase activation and apoptosis [6]. This process is inhibited by Trx. In a clinical point of view, it has been reported that serum Trx level is upregulated in the serum of the patients with chronic hepatitis [7].

In summary, we present evidence showing that TAA induces apoptosis of hepatocytes in the liver and that Trx inhibits TAA-induced apoptotic liver injury. This qualifies Trx as a promising gene therapy and a drug candidate for the treatment of acute hepatitis caused by virus infection and alcohol.
